# Genome-Wide Association Studies to Improve Wood Properties: Challenges and Prospects

**DOI:** 10.3389/fpls.2018.01912

**Published:** 2018-12-21

**Authors:** Qingzhang Du, Wenjie Lu, Mingyang Quan, Liang Xiao, Fangyuan Song, Peng Li, Daling Zhou, Jianbo Xie, Longxin Wang, Deqiang Zhang

**Affiliations:** ^1^Beijing Advanced Innovation Center for Tree Breeding by Molecular Design, Beijing Forestry University, Beijing, China; ^2^National Engineering Laboratory for Tree Breeding, College of Biological Sciences and Technology, Beijing Forestry University, Beijing, China; ^3^Key Laboratory of Genetics and Breeding in Forest Trees and Ornamental Plants, Ministry of Education, College of Biological Sciences and Technology, Beijing Forestry University, Beijing, China

**Keywords:** GWAS, omics, functional genomics, wood formation, systems biology

## Abstract

Wood formation is an excellent model system for quantitative trait analysis due to the strong associations between the transcriptional and metabolic traits that contribute to this complex process. Investigating the genetic architecture and regulatory mechanisms underlying wood formation will enhance our understanding of the quantitative genetics and genomics of complex phenotypic variation. Genome-wide association studies (GWASs) represent an ideal statistical strategy for dissecting the genetic basis of complex quantitative traits. However, elucidating the molecular mechanisms underlying many favorable loci that contribute to wood formation and optimizing GWAS design remain challenging in this omics era. In this review, we summarize the recent progress in GWAS-based functional genomics of wood property traits in major timber species such as *Eucalyptus*, *Populus*, and various coniferous species. We discuss several appropriate experimental designs for extensive GWAS in a given undomesticated tree population, such as omics-wide association studies and high-throughput phenotyping technologies. We also explain why more attention should be paid to rare allelic and major structural variation. Finally, we explore the potential use of GWAS for the molecular breeding of trees. Such studies will help provide an integrated understanding of complex quantitative traits and should enable the molecular design of new cultivars.

## Introduction

Wood, the secondary xylem of long-lived perennial plants, is produced via cell division from the vascular cambium, cell expansion, cell wall thickening, programmed cell death, and heartwood formation (Plomion et al., 2001; Mellerowicz and Sundberg, 2008). In general, the chemical and ultrastructural properties of wood depend on the components of the secondary cell walls, allowing wood to fulfill highly specialized functions that are essential for tree growth and development (Du et al., 2013a; Mizrachi and Myburg, 2016). Wood also represents a major carbon sink that plays a crucial role in carbon cycling in the terrestrial ecosystem, serving as an important renewable resource for the production of lumber, pulp, paper, and biofuels (Mellerowicz and Sundberg, 2008).

Progress has recently been made toward modifying the major wood biopolymers (i.e., lignin and cellulose) in several model plants (Robischon et al., 2011; Chen et al., 2014; Ye and Zhong, 2015), but much remains to be explored about the biosynthetic machinery of the chemistry and ultrastructure of wood cell walls. Most studies to date indicate that secondary wall biosynthesis needs the coordinated activity of transcriptional networks in regulating the diverse metabolic pathways involving polysaccharides and lignin biopolymers (Coleman et al., 2009; Mewalal et al., 2014). This intricate biological process incorporates a diverse set of xylem-forming genes, most with unknown functions (Zinkgraf et al., 2017). The genetic architecture and functional mechanisms that directly affect wood properties have not yet been fully identified and dissected.

Numerous studies have examined the underlying genetic variation in complex polysaccharides (alpha cellulose, hemicelluloses, and holocellulose), lignin (insoluble, soluble, syringyl, and total lignin), cell wall sugars (arabinose, glucose, mannose, and xylose), and ultrastructural traits (average density, crystallinity, fiber length, and microfiber angle) in *Eucalyptus, Populus*, and various coniferous species using forward genetic approaches, such as quantitative trait loci (QTLs) (Sewell et al., 2000; Novaes et al., 2009; Thumma et al., 2010; Yin et al., 2010) and candidate gene-based association mapping (Thumma et al., 2009; Dillon et al., 2010; Wegrzyn et al., 2010; Beaulieu et al., 2011; Du et al., 2013b; Guerra et al., 2013; Porth et al., 2013). However, the QTLs, single nucleotide polymorphism (SNP) loci, and candidate genes identified to date explain only a small proportion of the genetic variation of wood components. Genes cannot work in isolation; instead, multiple genes within complex biological pathways are often jointly involved in phenotypic variation (Du et al., 2015; Zinkgraf et al., 2017). Therefore, a more holistic research approach encompassing whole genome variation must be taken to understand and improve wood property traits.

Given the high genetic diversity, almost undomesticated status, rapid decay of linkage disequilibrium (LD), and minimal genetic structure of forest tree populations, such populations should represent ideal systems for conducting association studies and breeding using molecular marker-assisted selection (MAS). LD is fundamentally important for any genome-wide association study (GWAS) when genotyping does not cover all sequence variants in a genome. Indeed, the advantage of a low extent of LD is that once an association is detected, it is likely that this marker is physically close to the causal variant (likely within the gene itself) or is even the causal variant (Ingvarsson et al., 2016). Dissection of genome–phenotype associations through GWAS in a variety of systems is thus expected to efficiently bridge the gap between QTLs and causal genes in most forest trees (Porth et al., 2013), thanks to the currently available large populations and high-throughput sequencing technology. Here, we (1) summarize the recent progress in functional genomics of wood property traits via GWAS; (2) discuss the statistical methods and experimental designs needed to improve the use of GWAS in trees; and (3) explore opportunities for the use of GWAS in the molecular breeding of trees.

## Recent Progress in GWAS for Exploration of Wood Property Traits

Trees have a wide geographical distribution and large wild population sizes and thus exhibit diverse responses to environmental changes. Association mapping principally exploits evolutionary recombination at the natural population level. Thus, a collection of cultivars/natural individuals with unknown ancestry and newly designed nested association mapping (NAM) populations are often used for association analysis in trees. The availability of fast, accurate estimation methods for variance components is a prerequisite for performing GWAS. Yu et al. (2006) proposed a mixed linear model (MLM) method for better controlling population structure and the imbalanced kinships among various individuals (Pritchard et al., 2000). Genome-wide rapid association analysis by mixed model and regression (GRAMMER) was subsequently developed to roughly estimate random effects. Unlike approximate estimation models, the efficient mixed-model association (EMMA) matrix is a method for more accurately estimating the genetic and residual variance of a population, and therefore, may speed up the calculation process (Kang et al., 2008). EMMA eXpedited (EMMAX) and Population Parameters Previously Determined (P3D) are two other estimation methods that reduce the need for computational processing (Kang et al., 2010; Zhang et al., 2010). Factored spectrally transformed linear mixed models (FaST-LMM) (Lippert et al., 2011) and genome-wide efficient mixed-model association (GEMMA) (Zhou and Stephens, 2012) methods were subsequently developed. The advantage of these approaches is that they allow variance components to be directly estimated. Meta-GWAS and Joint-GWAS are used for obtaining higher statistical power in analyses of multiple tree populations (Wu et al., 2016; Müller et al., 2018).

Next-generation sequencing (NGS) and SNP arrays have opened up new possibilities for obtaining almost all SSRs and SNPs variants within a gene space or even within an genome-wide scan (Evans et al., 2014; Ingvarsson et al., 2016). Moreover, NGS has enabled genome-wide discovery of structural variation, insertion/deletions (InDels), and copy number variants (CNV) (Marroni et al., 2014), in a growing number of whole-genome resequencing studies in several model tree species (Evans et al., 2014; Silva-Junior and Grattapaglia, 2015; Wang J. et al., 2015; Wang et al., 2016; Du et al., 2016). These GWAS have yielded important insights into the genetic basis of complex quantitative traits in woody species, primarily focusing on wood compositions and wood property traits (Table 1) (Cappa et al., 2013; Porth et al., 2013; McKown et al., 2014; Allwright et al., 2016; Lamara et al., 2016; Gong et al., 2017; Resende et al., 2017; Zinkgraf et al., 2017).

**Table 1:** Summary of genome-wide association studies (GWAS) of wood property traits in main timber species.

Phenotype	Species	Population	Sample size	No. of markers	Method	Reference
Growth and wood properties	*Eucalyptus globulus*	Families and bulk collections	303	7,680 [Diversity Array Technology markers (DArT)]	General linear model (GLM) and unified mixed model (UMM)	Cappa et al., 2013
Wood density, stiffness, microfibril angle, and ring width	*Picea glauca*	Open-pollinated families	1694	7434 (SNPs)	Mixed linear model (MLM)	Lamara et al., 2016
16 wood chemistry/ultrastructure traits	*Populus trichocarpa*	Unrelated individuals	334	29,233 (SNPs)	GLM	Porth et al., 2013
Lignin percentage, Lignin S:G ratio, 5-carbon sugars, and 6-carbon sugars	*Populus deltoides*	Unrelated individuals	391	334,679 (consensus SNPs), 185,526 (Common SNPs), 76,804 (functional SNPs)	Single-variant and multiple-variant associations on GLM	Fahrenkrog et al., 2017
Basic wood density (BWD), bleached pulp, pulp yield (SPY), and pulp bleaching content	*Eucalyptus grandis × Eucalyptus urophylla*	Hybrid breeding population	768	24 806 (SNPs)	GWAS and regional heritability mapping	Resende et al., 2017
17 wood-quality traits	Norway spruce	Mother trees	517	178101 (SNPs)	Multilocus LASSO penalized regression	Baison et al., 2018
Seven wood properties	*Populus tomentosa*	Unrelated individuals	435	5,482 (InDels)	MLM and Kempthorne model	Gong et al., 2017


*Diversity Array Technology (DArT) markers*.

Porth et al. (2013) performed the first GWAS for key wood chemistry and ultrastructure traits in a population of 334 unrelated black cottonwood (*Populus trichocarpa*) individuals and found 141 significant SNPs associated with cell wall traits. Only 40% of these associations involve genes previously known to function in wood formation (Wegrzyn et al., 2010). For example, a synonymous SNP within the *FRA8* ortholog can explain 21.0% of the total genetic variance of fiber length, suggesting the GWAS could provide insight into the genetic basis of wood traits. Further integration of datasets from transcription factor binding, transcriptome profiling, and GWAS experiments in *Populus* provided an effective means of annotating conserved gene co-expression modules under diverse environmental conditions (Zinkgraf et al., 2017). The combinatorial utility of QTL and chromosome-wide association mapping enabled the authors to identify six genes (encoding the transcription factors ANGUSTIFOLIA C-terminus Binding Protein [CtBP] and KANADI, a Ca^2+^-transporting ATPase, an amino acid transporter, the copper transporter ATOX1-related, and a protein kinase) of functional relevance to cell wall recalcitrance in *Populus* (Muchero et al., 2015). These putative causal regulators may be utilized for selective breeding of trees.

These GWAS have largely focused on the common genetic variants using MLM-based methods. Identifying all functional variants that contribute to certain phenotypes has been infeasible. However, novel statistical models that combine the joint genetic effects of all variable loci at the whole-genome level have recently become the most popular types of models, allowing breeders to use genomic information to advance breeding programs (Yang N. et al., 2014; Resende et al., 2017; Xiao et al., 2017). For example, regional heritability mapping (RHM), has been proposed that provides heritability estimates for sequence segments with common or rare genetic effects (Nagamine et al., 2012). RHM was quite powerful for the detection of true QTLs in 768 hybrid *Eucalyptus* trees (Resende et al., 2017), suggesting that complex traits in *Eucalyptus* are controlled by multiple allele variants with rare effects.

Regardless of the statistical method utilized, the availability of numerous samples is especially important for detecting associated small-effect loci. Fahrenkrog et al. (2017) conducted GWAS for wood composition traits and detected a combination of common and rare SNPs by targeted resequencing of 18,153 genes in 391 unrelated *Populus deltoides* individuals. The results indicated that low-frequency SNPs associated with several bioenergy traits, suggesting that both common and rare variants must be considered in order to show a comprehensive picture of the genetic dissection of complex traits (Resende et al., 2017). Non-SNP allelic variation is another frequently used explanation for “missing heritability” of complex traits. Gong et al. (2017) utilized InDel-based GWAS to detect the causal variants underlying growth and wood properties in 435 unrelated *Populus tomentosa* accessions and identified regulatory InDels with an average of 14.7% phenotypic variance explained. The higher contribution of InDels to phenotypic variance compared to SNP loci, with a median of *c.* 5% explained, supports the notion that the InDels represent an effective marker system for MAS.

Genome-wide association study might also be feasible for coniferous species, even though their genomes are generally very large (often as much as 20 Gb). Uchiyama et al. (2013) examined the potential of performing GWAS in conifers using 367 unrelated plus trees of *Cryptomeria japonica* D. Don and identified six novel QTLs that were significantly associated with variation in wood property traits and the quantity of male strobili. All six SNPs were identified in sequences sharing similarity with known genes, such as genes encoding microtubule-associated protein RP/EB family members and a CLIP-associated protein. A xylem co-expression network was reconstructed in white spruce (*Picea glauca*) based on 180 wood-associated-genes, and the network hubs of several known NAC and MYB regulators, which were found using integrated GWAS and co-expression networks (Lamara et al., 2016). The genome sequence for Norway spruce (*Picea abies*) (Nystedt et al., 2013) provides a basis for functional multi-locus GWAS of wood properties in this species. A recent study used a multi-locus LASSO penalized regression method to identify 39 candidate genes involved in the formation of both early and late wood, as well as dynamic processes of juvenility, which could be useful for explaining the temporal regulation on secondary growth (Baison et al., 2018).

Over the past 5 years, new whole-genome resequencing and emerging GWAS approaches that have been used to dissect ecologically relevant traits and wood traits in forest trees (Evans et al., 2014; Ingvarsson et al., 2016). Zhang et al. (2018) recently performed GWAS and expression QTL (eQTL) analyses of 917 *P. trichocarpa* accessions and found that *hydroxycinnamoyl-CoA:shikimate hydroxycinnamoyl transferase 2* (*PtHCT2*) controlled chlorogenic acid (CGA) and partially characterized metabolite levels, providing novel insights into omics-based inference of gene function in trees. Such practical applications of GWAS require the development of novel statistical methods, databases, and experimental designs for forest trees.

## Future Applications of GWAS to Analyze Complex Quantitative Traits in Trees

Complex phenotypic variations, such as wood formation in long-lived tree populations, involve a series of dynamic biological processes orchestrated in a precise, quantitative manner, including the transcriptional and translational regulation and the flux of metabolic intermediates of diverse biochemical pathways (Mizrachi and Myburg, 2016). We take advantage of GWAS to explore the single-marker effects, pleiotropic relationships, genetic interactions among these loci, and their interactions with different environmental factors (Ingvarsson et al., 2016; Plomion et al., 2016). Thus, based on recent advances in GWAS for identification of loci affecting wood property traits, we will discuss several statistical methods and experimental designs that could facilitate the extensive application of GWAS in a given undomesticated tree population.

### Drive Omics-Wide Association Study (OWAS) in Trees

**FIGURE 1:**
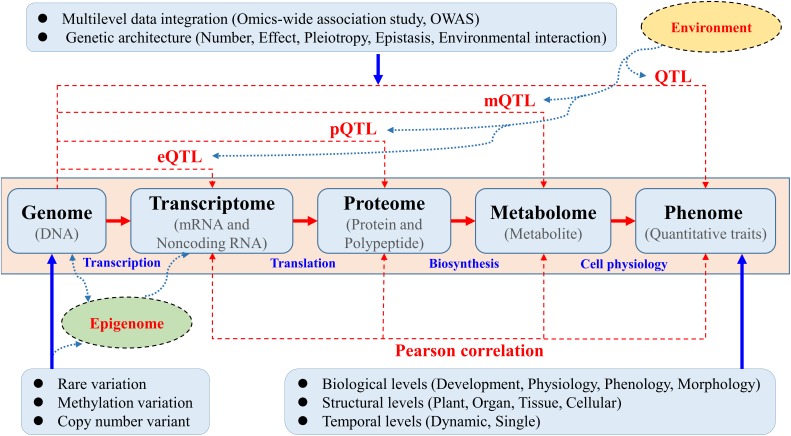
Multiple omics-wide association mapping of complex phenotypes aimed at constructing systems maps in trees. Systematic OWAS was used to map interactive expression QTLs (eQTLs), protein QTLs (pQTLs), and metabolite QTLs (mQTLs) underlying these different profiles for complex quantitative traits. Traditional genotype–phenotype associations to detect the underling QTLs were calculated via GWAS. Systems OWAS mapping treats phenotypic formation as a dynamic system composed of many interactive, functional elements at different levels. In this systems model, mRNA and non-coding RNA expression, protein and polypeptide levels, and metabolite levels are measured using different methods. These data could be integrated with epigenomic and environment interaction data to construct a complete biological working model.

Knowledge of the genetic basis and hierarchical interactions of higher-order variation at an intermediate level, such as transcriptomic, proteomic, and metabolomic variation ranging from genotypic to phenotypic, has proven to be a great resources as “molecular phenotypes” will be crucial for dissecting the functional pathways underlying complex quantitative traits in trees (Figure 1). The joint use of “-omics”-wide data will help disclose candidate genes and functional pathways underlying target traits beyond GWAS (Feltus, 2014; Mizrachi and Myburg, 2016). The comprehensive reconstruction of global biochemical networks across multiple omic layers would entail the use of omics-wide association study (OWAS) techniques proposed based on both multi-omic measurements and computational data integration. A systematic OWAS approach must therefore be adopted to map interactive eQTLs, protein QTLs (pQTLs), and metabolite QTLs (mQTLs) underlying these different profiles for quantitative traits (Figure 1). The opening of this new OWAS era in trees will allow us to identify minor QTLs that are masked by major loci (Luo, 2015; Xiao et al., 2017).

As a prerequisite, precise sequencing and rigorous annotation of holistic reference genomes are needed for large heterozygous tree genomes, which remains challenging. Work is also needed to refine new sequencing technologies and to develop relevant bioinformatics approaches, which would lay the foundation for identifying genome-wide allele variation and performing massively parallel RNA sequencing (RNA-seq) of 1000s of genotypes (Alonso-Blanco and Méndez-Vigo, 2014). In particular, non-coding RNAs constitute a specific component of the transcriptome worthy of attention, which could provide new insights into the regulatory mechanisms underlying eQTLs (Zhou et al., 2015; Plomion et al., 2016; Quan et al., 2018). In this new omics era for trees, many challenges remain. For example, if differences between tissues and developmental stages were not considered, conventional mapping methods would not be suitable for identifying new higher-level omics variation.

### Development of High-Throughput Phenotyping Technologies

In addition to obtaining complete genome sequence information, the improvement of phenotyping methods and the availability of high-quality trait data are also priority areas for successful GWAS in forest trees (Li Z. et al., 2014; Du et al., 2016). Trees are usually large and have a long lifespan, making it difficult to perform phenotyping in outdoor test plantations on the biological, structural, and temporal levels (for example, tree biomass, wood properties, and biotic and abiotic responses to stress; Figure 1). The conventional procedures used for phenotyping of tree populations, which represent a “phenotyping bottleneck” (Furbank and Tester, 2011). The highly dynamic nature of genes, proteins, and metabolite levels along a temporal or developmental trajectory, point to the need for careful experimental design and sampling conditions to ensure that the samples (Alonso-Blanco and Méndez-Vigo, 2014) will provide relevant insights about the phenotype of interest.

More attention should focus on how to develop high-throughput phenotyping (HTP) technologies to increase the precision of time-series data for functional plant traits. The emerging plant HTP platform uses a variety of imaging methods including visible imaging, imaging spectroscopy, thermal infrared imaging, fluorescence imaging, 3D imaging, and tomographic imaging to collect data for quantitative studies of complex traits related to plant growth, yield, adaptation, morphological and physiological traits (Li L. et al., 2014; Gómez-Candón et al., 2016; Yendrek et al., 2017).

Such a computational ecosystem provides tremendous opportunities for solving foundational problems in predictive phenomics and accelerating breeding efforts (Singh et al., 2016; Shakoor et al., 2017). For example, high-throughput 3D imaging can be used to map QTLs underlying variation in root architecture and seed traits in herbaceous plants (Moore et al., 2013; Topp et al., 2013). Gómez-Candón et al. (2016) used thermal IR data to characterize individual apple tree responses to drought, and it can identify genotypic variation with differential phenotypic responses to water limitation. Plant phenomics facilities have been opening worldwide (Brown et al., 2014). Careful consideration of the appropriate sensors and HTP time points for field traits is important for data collection in long-lived woody species. For example, RGB color camera networks can be deployed to collect images continuously over the course of periods to assess biomass, growth rate, and disease progression. In this emerging era of phenomics, a tree HTP platform utilizing high-performance computing software and measurement hardwire is needed to collect multi-dimensional phenotyping data for different environments over long periods of time. Therefore, the resolution and precision of phenotyping systems should be higher for long-lived trees than the HTP systems developed for annual herbaceous plants (Meijón et al., 2014; Slovak et al., 2014; Yang W. et al., 2014).

### Increase the Focus on Rare Variation and Major Structural Variation

Over the past few years, population-scale resequencing of the human genome has enabled more comprehensive analysis of allelic effects on certain complex diseases. Some rare variants (i.e., minor allele frequency [MAF] < 1% or 5%) are likely to be more extreme than common variants for susceptibility to diseases with high and low heritability (Genomes Project et al., 2010; Nelson et al., 2012; Tennessen et al., 2012). Most association studies in plant species have typically removed rare variants due to the limited power of statistical approaches for detecting their contribution to the phenotypic variation of complex traits (Porth et al., 2013; Du et al., 2015).

Progress has been made in several crops and model plants. For example, Wang S. et al. (2015) exploited rare variation mapping to discover a TCP transcription factor gene vital for tendril development in cucumber (*Cucumis sativus* L.). Xing et al. (2015) found that a low-frequency SNP within *Brachytic2* can moderately increase yield and reduce stem height in maize. The genomes of woody species have not experienced reductions in genetic diversity due to domestication, and rare variants are abundant among tree genomes but less tractable in GWAS. Such abundant rare variants are actually important for explaining the missing heritability of complex traits (Resende et al., 2017).

Rare beneficial alleles are usually employed in current tree breeding, which once proven useful, undergo selective sweep and become fixed in all major cultivars. Balancing samples across population subdivisions and increasing the sample size can homogenize allele frequencies and elevate globally rare variants to common markers in some subpopulations. In addition, the use of multiple bi-parental crosses can break up population structure, which could increase the power of detecting multiple rare alleles that underlie natural variation. The promising approach of joint mapping with association panels and multiple bi-parental crosses in trees is also valuable for identifying low-frequency or small-effect alleles (Du et al., 2016). Decreases in sequencing costs and improvements in genotyping technologies have promoted the use of exceptionally large diverse populations to identify and analyze rare variants. Other statistical models and approaches, such as RHM (Nagamine et al., 2012) and the SKAT package for estimating the joint effect (Fahrenkrog et al., 2017), should be used to detect and dissect rare variation in tree populations.

Advances in sequencing technology and bioinformatics algorithms offer the potential to test previously undetected structural variation within re-sequencing populations of a species (Xiao et al., 2017). In contrast to the currently known SNPs and SSRs, CNVs can be categorized into deletions, duplications, and insertions that have not been given much attention in tree species (Feltus, 2014; Ingvarsson et al., 2016). Hence, investigating the roles, structures, and functions of CNVs as both biomarkers for mapping efforts and potential functional variants in woody plants should provide vital findings about phenotypic variation (Marroni et al., 2014). The presence of high levels of heterozygosity in perennial forest trees reduces the power of CNV detection. To address this issue, the precision of genome sequencing should be enhanced and the resequencing coverage depth should be increased, which would contribute to fine read-depth-based CNV identification.

### Dissect the Often-Ignored Epistasis Effects in GWAS

Genetic variation of quantitative traits is classified into additive, dominant, and epistatic effects, which are conferred by numerous genes/alleles in the multiple biological networks (Du et al., 2015). It is likely that non-additive interactions between separate mutations (epistasis) often reflect the missing heritability and lack of inter-population validation of causal variants (Manolio et al., 2009). However, detecting locus-locus epistasis is challenging experimentally, statistically, and computationally due to the large number of complex interactions to be dissected in GWAS (Mackay, 2014). Several association algorithms, such as BiForce (Gyenesei et al., 2012), genome-wide interaction studies (GWIS, Goudey et al., 2013), the Kempthorne model (Mao et al., 2007), and FastEpistasis (Schüpbach et al., 2010) make it possible to test for two-way epistasis between all locus pairs. Indeed, testing for pairwise epistasis would shed light on genetic architecture.

**FIGURE 2:**
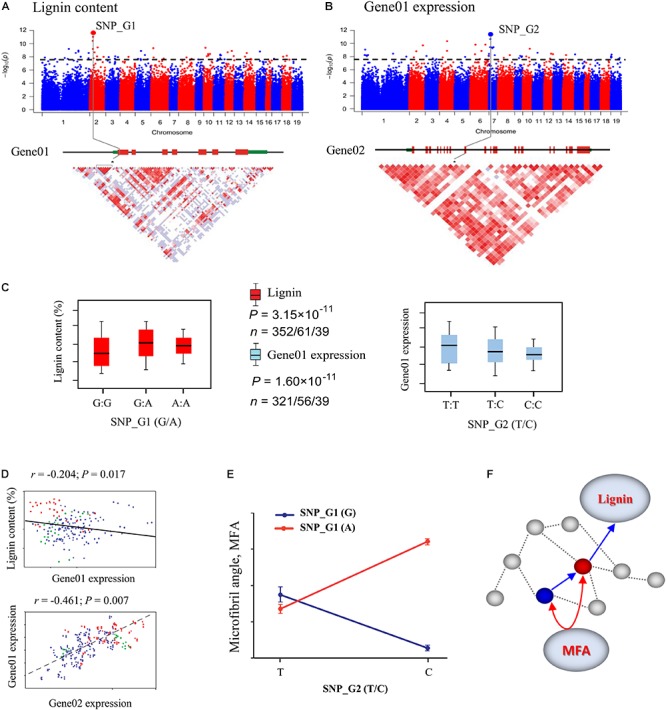
Identification of single marker effects and epistasis for wood traits using the systems genetics integrative framework. **(A)** Manhattan plots displaying genome-wide association study (GWAS) results for lignin content. The most significant SNP (SNP_G1) associated with lignin content was identified, which is located in exon 1 of a gene (Gene01). **(B)** GWAS results for Gene01 expression using express QTL (eQTL) methods. The most significant SNP (SNP_G2) associated with Gene01 expression was identified, which is located in exon 9 of a gene (Gene02). The x-axis shows the chromosome positions and the y-axis shows significance expressed as –log_10._ The dotted horizontal line depicts the Bonferroni-adjusted significance threshold (0.05/n). Three linked genes are shown at the bottom (red rectangle, coding sequences; black line, introns; green rectangle, 5′ and 3′ untranslated regions). **(C)** Box plot for lignin content trait (red) and expression of Gene01 (sky blue) plotted as an effect of genotypes at the lead SNP. The horizontal line represents the mean and the vertical lines mark the range from the 5th and 95th percentile of the total data. **(D)** Plots of correlation between each pair for lignin content, Gene01 expression, and Gene02 expression level among the genotype classes. The *r*-value is based on the Pearson correlation coefficient. The *P*-value was calculated using the *t* approximation. **(E)** Pairwise interactions between SNP_G1 and SNP_G2 control Microfibril angle (MFA) with different genotypic combinations at the two loci. **(F)** These GWAS and eQTL data were used to construct the expression network shown in the figure. Gene02 (blue dot) is located at the identified eQTL significance peak and is the network hub that affects the downstream Gene01 (red dot) to affect lignin content. Gene02 can also interact with Gene01 in the network, which is associated with MFA.

Additionally, dissecting the genome-wide genetic basis of exhaustive gene/allele epistasis using different populations could provide insight into the roles of epistasis in response to local adaptation in trees (Du et al., 2018). Once a gene–gene interaction network is constructed using GWAS (Figure 2), it can be validated as communities of genes (gene modules) that interact strongly within the complex network using RNA profiling, molecular biology, and biochemistry (Mackay, 2014; Gong et al., 2017). Quantitative traits controlled by multiple gene networks are often regulated by higher-order gene–gene interactions that are too complex to be analyzed by standard two-way epistatic tests (Du et al., 2015). In the future, more attention should focus on the pleiotropic effects of epistasis on gene expression, metabolites, and development in different geographical populations. Such knowledge would provide insights into the mechanisms and functions of the gene networks underlying plant traits.

## Perspectives for Post-GWAS and Molecular Breeding in Trees

A deeper knowledge of the genetic basis of complex traits, such as wood properties, can be achieved through the integrated use of diverse statistical models and experimental tools. Systems genetics could be used to understand the molecular mechanisms of candidate genes underlying traits of interest. Some promising studies demonstrate the advantages of this approach across a tree’s lifespan (Feltus, 2014; Ingvarsson et al., 2016; Plomion et al., 2016). However, functional validation and annotation of causal loci remain challenging, especially because most loci detected by GWAS are located in intergenic regions or are not canonical components of previously identified pathways (Civelek and Lusis, 2013; Xiao et al., 2017). Given the growing number of genomic variations mapped by GWAS for a given trait, a systems biology approach may be needed to validate the function of these alleles using holistic data that provide evidence for causality. Family-based accession intercrosses using different alleles could be performed to clearly determine whether an allelic variant of a specific gene is causal for the observed natural trait variation (Du et al., 2016). Promising advances in genetic transformation and genomic technologies as well as statistical and computational methods may help to address these issues.

Tree MAS breeding may depend on the use of a pleiotropic allele or a favorable combination of alleles for multiple traits of interest (Lamara et al., 2016), such as the MYB transcription factor and LncRNA genes identified for lignin and polysaccharides traits (Zhou et al., 2017; Quan et al., 2018). Thus, it is particularly important to link specific alleles to corresponding traits. High-throughput whole-genome sequences should enable the use of alternative breeding approaches, including genomics-assisted selection (GAS) and genome selection (GS) breeding strategies, as well as genome-based phenotypic prediction and design breeding using rare, recessive, large-effect mutations (Resende et al., 2012; Evans et al., 2014; Varshney et al., 2016). Specifically, using GWAS tools, it will be possible to identify many more causative genes and their roles in multiple linked traits, highlighting the potential of GWAS for systematically uncovering the balanced regulation between these traits and identifying the key hub genes that link them (Feltus, 2014; Lamara et al., 2016). However, QTL and GWAS information has not yet been successfully used for the molecular breeding of perennial trees.

High-throughput GWAS combined with precision genome editing has huge potential for combining currently available causal alleles in the gene pool (Zhou et al., 2015; Xiao et al., 2017). This GAS strategy could be used to accelerate the improvement of wood traits, as well as plant growth and stress resistance. In addition, whole-genome prediction of hybrid performance will be very important for tree breeding design, as heterosis has been successfully exploited for many major forest species. Selection and crosses based on integrated genomics approaches, as well as genetic modification via transformation, could potentially be used to develop new and superior cultivars.

## Author Contributions

DZhang planned the review. QD, DZhou, WL, MQ, and LX designed the figures and revised the review. QD, FS, PL, JX, and LW collected the data. QD wrote the paper. All authors read and approved the manuscript.

## Conflict of Interest Statement

The authors declare that the research was conducted in the absence of any commercial or financial relationships that could be construed as a potential conflict of interest.
